# Virulent *Mycobacterium bovis* Beijing Strain Activates the NLRP7 Inflammasome in THP-1 Macrophages

**DOI:** 10.1371/journal.pone.0152853

**Published:** 2016-04-04

**Authors:** Yang Zhou, Syed Zahid Ali Shah, Lifeng Yang, Zhongqiu Zhang, Xiangmei Zhou, Deming Zhao

**Affiliations:** 1 National Animal Transmissible Spongiform Encephalopathy Laboratory, Key Laboratory of Animal Epidemiology and Zoonosis of Ministry of Agriculture, College of Veterinary Medicine and State Key Laboratory of Agrobiotechnology, China Agricultural University, Beijing 100193, China; 2 Veterinary Bureau, Ministry of Agriculture of the People’s Republic of China, Beijing 100125, China; University of Maryland, UNITED STATES

## Abstract

*Mycobacterium bovis* is the causative agent of tuberculosis in a wide range of mammals, including humans. Macrophages are the first line of host defense. They secrete proinflammatory cytokines, such as interleukin-1 beta (IL-1β), in response to mycobacterial infection, but the underlying mechanisms by which human macrophages are activated and release IL-1β following *M*. *bovis* infection are poorly understood. Here we show that the ‘nucleotide binding and oligomerization of domain-like receptor (NLR) family pyrin domain containing 7 protein’ (NLRP7) inflammasome is involved in IL-1β secretion and caspase-1 activation induced by *M*. *bovis* infection in THP-1 macrophages. NLRP7 inflammasome activation promotes the induction of pyroptosis as well as the expression of tumor necrosis factor alpha (TNF-α), Chemokine (C-C motif) ligand 3 (CCL3) and IL-1β mRNAs. Thus, the NLRP7 inflammasome contributes to IL-1β secretion and induction of pyroptosis in response to *M*. *bovis* infection in THP-1 macrophages.

## Introduction

*Mycobacterium bovis*, a member of the *M*. *tuberculosis* complex, is the etiological agent of bovine tuberculosis which is estimated to infect more than 50 million cattle per annum with concomitant economic losses of approximately $3 billion worldwide [1]. *M*. *bovis* is also responsible for a proportion of human tuberculosis (TB) cases, and can be transmitted from human to human. About 2.8% of all human TB cases in Africa are caused by *M*. *bovis*, and human *M*. *bovis* infection accounts for 7.6% of cases in Mexico which also contributes to the disease incidence in the United States, although overall incidence in the Americas is low [2]. This huge zoonotic risk imposes limitations on the potential for control [3, 4].

Macrophages are considered the first line of host defense against invasive microbes. Upon infection, they initiate inflammatory responses by releasing cytokines and chemokines, such as IL-1β, IL-18, TNF-α, and CCL3. Among these, IL-1β is a potent mediator of antimicrobial responses. It contributes to the maturation of mycobacterial phagosomes into phagolysosomes, which enhances mycobacterial elimination by macrophages. Inhibition of IL-1β activity by neutralizing antibody or siRNA increases intracellular mycobacterial survival [5]. Conversely, adding exogenous IL-1β markedly inhibits their survival [6]. IL-1β activity is tightly controlled at the levels of expression, maturation, and secretion [7]. It is initially synthesized as a precursor molecule, proIL-1β, in the cytosol in response to pathogen-associated molecular patterns (PAMPs), which are sensed by evolutionarily-conserved toll-like receptors. Its maturation and secretion requires caspase-1 activation by multiprotein complexes known as inflammasomes [8]. The inflammasomes consist of a receptor protein, the adaptor apoptosis-associated speck-like protein containing a caspase-activation recruitment domain (ASC), and caspase-1. Receptor proteins include NLR family pyrin domain containing 3 protein (NLRP3) [9], absent in melanoma 2 (AIM2) [10], NLR family caspase-activation recruitment domain (CARD)-containing protein 4 (NLRC4) [11], and NLRP7, which is uniquely stimulated by microbial acetylated lipopeptides [12]. The majority of research on NLRP7 has been associated with hydatidiform mole, an abnormal human pregnancy with hyperproliferative vesicular trophoblast and no fetal development [13]. A recent study showed that some live and heat killed microbes, including *Mycoplasma* spp., *Staphylococcus aureus* and *Listeria monocytogenes*, activate the NLRP7 inflammasome. NLRP7 senses lipopeptides through its leucine-rich repeat (LRR) domain [12], and results in self-oligomerization to form an inflammasome scaffold through its nucleotide-binding and oligomerization (NACHT) domain. It interacts with ASC via homotypic pyrin domain interactions, recruiting procaspase-1 via the CARD domain of ASC. Procaspase-1 clustering leads to caspase-1 auto-activation and generation of active caspase-1, which cleaves inactive proinflammatory cytokines into their active forms.

Little is known about the protective role of the NLRP7 inflammasome against either *M*. *bovis* or *M*. *tuberculosis* in macrophages. *M*. *bovis* is an intracellular pathogen expressing and secreting lipoproteins [14–16] and we demonstrate here that *M*. *bovis* infection triggers NLRP7 inflammasome activation and induction of pyroptosis in human THP-1 macrophages.

## Materials and Methods

### Reagents

The following antibodies and reagents were purchased from the indicated suppliers: the mouse monoclonal antibody against NLRP7 used for immunofluorescence assay and rabbit polyclonal anti-AIM2 antibody, Santa Cruz Biotechnology; rabbit polyclonal anti-NLRP7 antibody used for western blotting, Pierce/Thermo Fisher Scientific; rabbit polyclonal anti-ASC antibody and rabbit polyclonal anti-NLRP3 antibody, Sangon Biotech, Shanghai, China; goat polyclonal anti-IL-1β antibody, R&D Systems; rabbit polyclonal anti-β-actin antibody, Proteintech, Wuchan, China; rabbit polyclonal anti-caspase-1 antibody, ProSci Incorporated; phorbol 12-myristate-13-acetate (PMA), Sigma-Aldrich; glycine, Beijing Solarbio Science & Technology Co., Ltd.; cytochalasin D, Cayman Chemical; and Z-YVAD-FMK, BioVision Incorporated.

### THP-1 cell culture and differentiation

THP-1 cells were obtained from American Type Culture Collection (Manassas, VA, USA) and maintained in RPMI 1640 medium (Gibco, Grand Island, NY, USA) containing 10% fetal bovine serum (FBS, Gibco). THP-1 cells were stimulated with PMA (5 ng/mL) to differentiate into macrophages for 2 days, after which the cells were washed three times with warm phosphate-buffered saline (PBS). Cells were then incubated in PMA-free culture medium and rested for a further 2 days.

### Bacterial culture and infection

Virulent *M*. *bovis* Beijing strain was obtained from the China Institute of Veterinary Drug Control, Beijing and grown in 7H9 Middlebrook media (BD Biosciences) supplemented with albumin-dextrose-catalase (ADC) enrichment solution and 0.05% Tween-80 (Difco) at 37°C. THP-1 macrophages were infected with *M*. *bovis* at a multiplicity of infection (MOI) indicated for 2 h and then washed three times with warm PBS to remove extracellular bacteria. The samples were harvested at the indicated time.

### Small interference RNA (siRNA) transfection

THP-1 macrophages were transfected with gene-specific siRNA pools to knock down NLRP7 or ASC. Human NLRP7-targeting siRNA oligonucleotides, ASC-targeting siRNA oligonucleotides and non-targeting control siRNA oligonucleotides were obtained from Shanghai GenePharma Co., Ltd (Table 1). THP-1 cells were differentiated and then incubated overnight in a 24-well plate. Prior to transfection, all medium was removed and 400 μL of fresh medium was added. Lipofectamine 3000 transfection reagent and siRNA were added into 100 μL of serum-free culture medium, and incubated for 10 min at room temperature. The resulting mixture was added drop-wise onto the cells and culture medium was replaced after 24 h.

**Table 1:** Sequences of ASC-targeting siRNA and non-targeting control siRNA.

Name	Sequence (sense, antisense)
NLRP7-targeting siRNA:	
Target Sequence 1:	GACGUCACUCUGAGAAACCAATT
	UUGGUUUCUCAGAGUGACGUCTT
Target Sequence 2:	GUCAGAGGGUCACAUGUUATT
	UAACAUGUGACCCUCUGACTT [12]
Target Sequence 3:	GUGUUCCUGGAGAAUUACATT
	UGUAAUUCUCCAGGAACACTT [12]
ASC-targeting siRNA:	
Target Sequence 1:	UCGCGAGGGUCACAAACGUTT
	ACGUUUGUGACCCUCGCGATT
Target Sequence 2:	UGCUGUCCAUGGACGCCUUTT
	AAGGCGUCCAUGGACAGCATT
Target Sequence 3:	GCAAGAUGCGGAAGCUCUUTT
	AAGAGCUUCCGCAUCUUGCTT
Non-targeting siRNA:	UUCUCCGAACGUGUCACGUTT
	ACGUGACACGUUCGGAGAATT

### Quantitative real-time PCR

Total RNA extraction was performed using RN28-EASYspin Plus Tissue/Cell RNA Kit (Aidlab Biotech, Beijing) and reverse transcription was performed using RevertAid First Strand cDNA Synthesis Kit (Thermo Fisher). Quantitative PCR was carried out in a Roche LightCycler480 II using TransStart Green qPCR SuperMix UDG (Beijing TransGen Biotech). Primers for NLRP7 were 5´-TAAGGAATGCGACTGTGAACATC-3´ forward and 5´-TGCTAACTCCGAGTCTTCTTCT-3´ reverse. Primers for NLRP3, AIM2, and GAPDH were the same as those used in our previous study [17].

### Western blotting

Cells were washed in PBS, and lysed in cold lysis buffer (Beyotime Institute of Biotechnology, China) for 20 min. Samples were centrifuged at 12,000 ×*g* for 20min and the supernatant was boiled for 10 min after addition of loading buffer (250 mM Tris-HCl pH 6.8, 10% SDS, 0.5% BPB, 50% glycerol, 0.5 M DTT). For detection of IL-1β and caspase-1 released into the culture medium, proteins were precipitated as described previously [18]. Aliquots were separated via SDS-PAGE and the proteins were transferred to PVDF membranes (Immobilon-PSQ, ISEQ00010, 0.2 μm). Blots were blocked by 5% non-fat milk in TBST (25 mMTris base, 137 mM sodium chloride, 2.7 mM potassium chloride and 0.05% Tween-20, pH7.4) for 1 h at room temperature, incubated with the indicated primary antibody overnight at 4°C and the corresponding HRP-labeled secondary antibody for 50 min at 37°C, and the signal detected using an enhanced chemiluminescence (ECL) detection kit (Bio-Rad, USA).

### Immunofluorescence

THP-1 cells were fixed with 4% paraformaldehyde for 10 min at room temperature. Following permeabilization with 0.1% Triton X-100 for 10 min, the cells were blocked with 1% BSA for 1 h, incubated with primary antibodies overnight at 4°C and secondary antibodies at 37°C for 1 h. Nuclei were stained with DAPI for 1 min. Finally, coverslips were mounted on slides, and the cells were imaged using confocal microscopy. Colocalization was quantified using ImageJ software.

### Lactate dehydrogenase (LDH) release assay

LDH release was measured using LDH Cytotoxicity Assay Kit (Cayman Incorporated) according to the manufacturer’s instructions.

### Statistical analysis

All assays were performed in three independent experiments and data were analyzed using GraphPad Prism 5.0 software and Student’s t test; p < 0.05 values were considered statistically significant.

## Results

### *M*. *bovis* infection induces caspase-1 activation and IL-1β secretion in THP-1 macrophages

**Fig 1:**
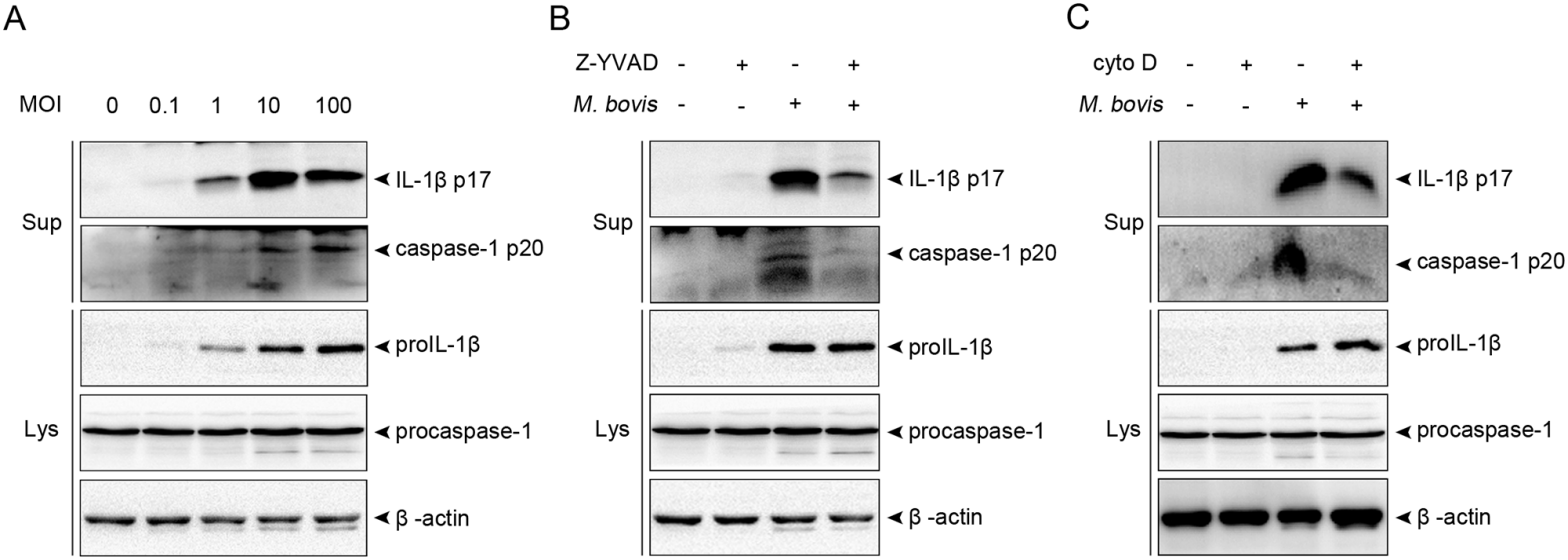
*M*. *bovis* triggers caspase-1 activation and IL-1β secretion in THP-1 macrophages. A. Cells were infected at the indicated MOIs and samples were harvested at 14 hpi. Culture supernatant was analyzed for IL-1β and caspase-1, and cell lysates were analyzed for proIL-1β, procaspase-1, and β-actin by immunoblotting. B. Cells were pretreated with 50 μM Z-YVAD-FMK for 1 h, and then infected with *M*. *bovis* at an MOI of 10 in the presence or absence of Z-YVAD-FMK. Culture supernatant and lysates were analyzed by immunoblotting. C. Cells were treated with 1 μg/mL cytochalasin D to block phagocytosis for 1 h, and then infected with *M*. *bovis* in the presence or absence of cytochalasin D. Culture supernatant and lysates were analyzed by immunoblotting. Abbreviations: Sup, culture supernatant; Lys, cell lysates; Z-YVAD, Z-YVAD-FMK; cyto D, cytochalasin D. Data from one representative experiment of three are presented.

We first examined whether *M*. *bovis* could induce caspase-1 activation and IL-1β secretion in THP-1 monocyte-derived macrophages at various MOIs (Fig 1A). Infection of THP-1 macrophages with *M*. *bovis* led to release of IL-1β into the supernatant in a dose-dependent fashion at MOIs ranging from 0.1 to 10, but IL-1β secretion was not enhanced further at an MOI of 100. Meanwhile, bacterial challenge resulted in increased production of proIL-1β, a precursor of IL-1β. *M*. *bovis* also induced caspase-1 maturation, as evidenced by increased levels of the cleaved p20 subunit in the supernatant, which directly correlated with MOI. To investigate whether capase-1 activation is required for IL-1β secretion induced by *M*. *bovis*, THP-1 macrophages were pretreated with Z-YVAD-FMK, a cell permeable inhibitor of caspase-1; this considerably reduced IL-1β secretion (but not proIL-1β production) upon *M*. *bovis* infection (Fig 1B). As *M*. *bovis* can be internalized by macrophages, the contribution of intracellular bacteria to IL-1β secretion and caspase-1 activation was examined. THP-1 macrophages were treated with cytochalasin D, a drug that inhibits actin polymerization and thus blocks phagocytosis of *M*. *bovis*. Substantial inhibition of caspase-1 activation and IL-1β release were observed following *M*. *bovis* infection (Fig 1C). Taken together, these data show that *M*. *bovis* induces IL-1β secretion in a caspase-1-dependent manner in THP-1 macrophages.

### *M*. *bovis* infection upregulates the expression of NLRP7 mRNA

**Fig 2:**
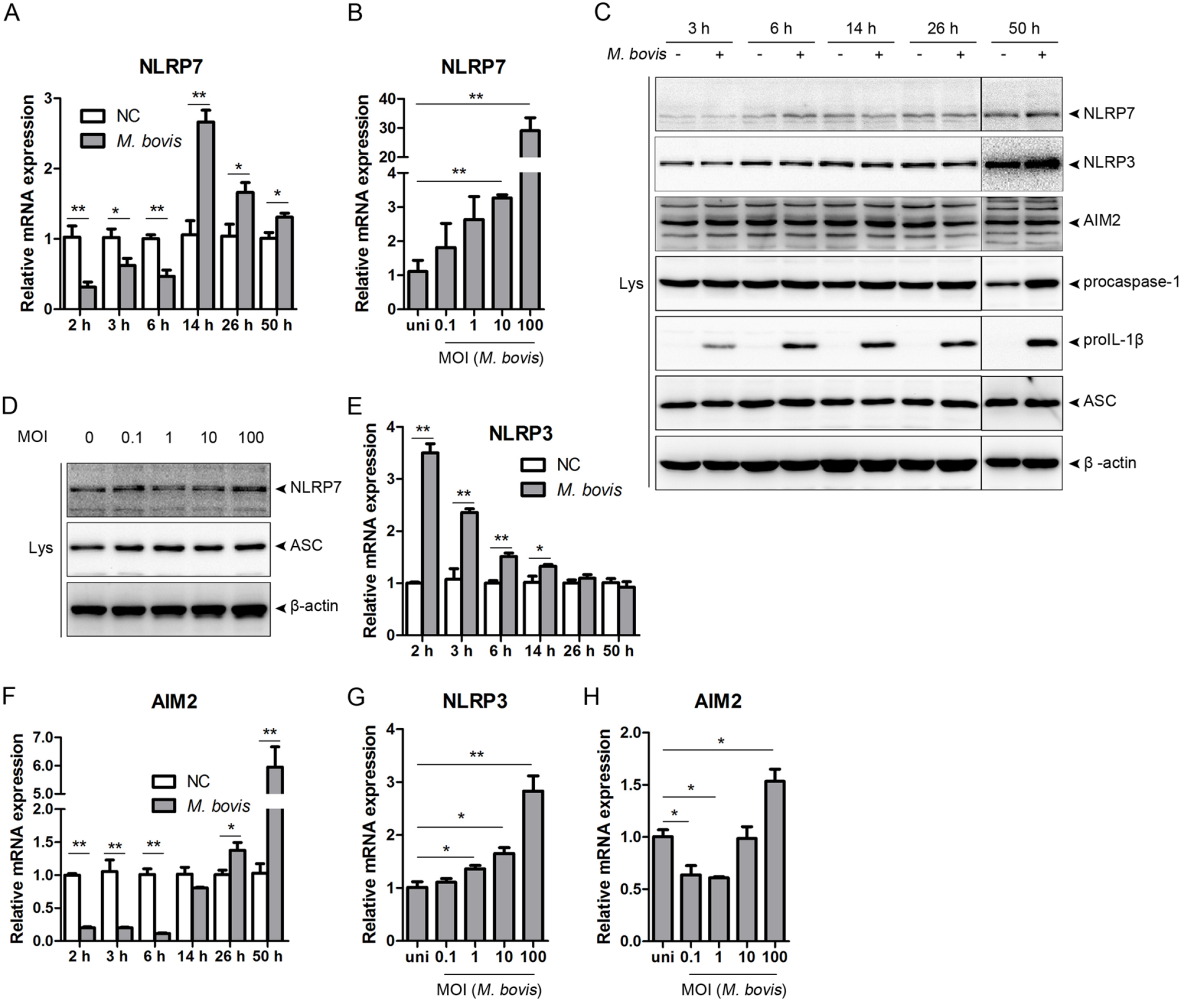
*M*. *bovis* infection leads to upregulation of NLRP7 mRNA expression. A, E, F. THP-1 macrophages were infected with *M*. *bovis* for the indicated times. Cell lysates were subjected to quantitative real-time PCR analysis. *0.01 <*P* < 0.05, **P < 0.01. B, G, H. Cells were infected with *M*. *bovis* at the indicated MOI. Lysates were harvested at 14 hpi, and subjected to quantitative real-time PCR analysis. C. Cells were infected with *M*. *bovis* for the indicated times, and supernatant and lysates were analyzed by immunoblotting. D. Cells were infected with *M*. *bovis* at the indicated MOI at 14 hpi, and supernatant and lysates were analyzed by immunoblotting.

The NLRP7 inflammasome is activated by a variety of microorganisms through the recognition of microbial acetylated lipopeptides. Since *M*. *bovis* expresses secreted and membrane-associated lipoproteins, such as MPB70/80, MPB83 [16], and P27 [15]. To explore whether the NLRP7 inflammasome is activated by *M*. *bovis*, we initially examined the time course of the mRNA expression of NLRP7 in THP-1 macrophages. Treatment with *M*. *bovis* significantly upregulated the expression of NLRP7 mRNA at 14 h post-infection (hpi). The levels increased to approximately 3-fold relative to negative control, and then decreased to 1.3-fold at 50 hpi (Fig 2A). Stimulation with *M*. *bovis* at different MOIs ranging from 0.1 to 100 revealed that NLRP7 was upregulated in a dose-dependent manner (Fig 2B). Although there was an increase in transcriptional level, infection with *M*. *bovis* at MOIs of 0.1 and 1 failed to produce a significant change. In spite of upregulation of NLRP7 at the mRNA level, there is no change at the protein level even at an MOI of 10 at 50 hpi (Fig 2C), or at an MOI of 100 at 14 hpi (Fig 2D). The AIM2 inflammasome is activated through the recognition of DNA during *M*. *bovis* infection [19, 20], and the NLRP3 inflammasome is thought to be activated after exposure to the secreted protein, ESAT-6 [21, 22]. To clarify the role of the AIM2 and NLRP3 inflammasomes in *M*. *bovis*-infected THP-1 macrophages, we quantified their expression following infection. There was a rapid induction of NLRP3 mRNA within 2 hpi, which then fell gradually in a time-dependent manner, reaching control levels at 26 hpi (Fig 2E). AIM2 mRNA increased as early as 26 hpi, and continued to increase at 50 hpi (Fig 2F). *M*. *bovis* infection failed to induce any changes in protein levels of NLRP3 and AIM2 (Fig 2C). Infections at various MOIs showed that *M*. *bovis*-induced upregulation of both NLRP3 and AIM2 was dose-dependent (Fig 2G and 2H). Thus, *M*. *bovis* induces upregulation of the mRNA level of NLRP7 besides NLRP3 and AIM2.

### The NLRP7 inflammasome contributes to caspase-1 activation and IL-1β secretion during *M*. *bovis* infection

**Fig 3:**
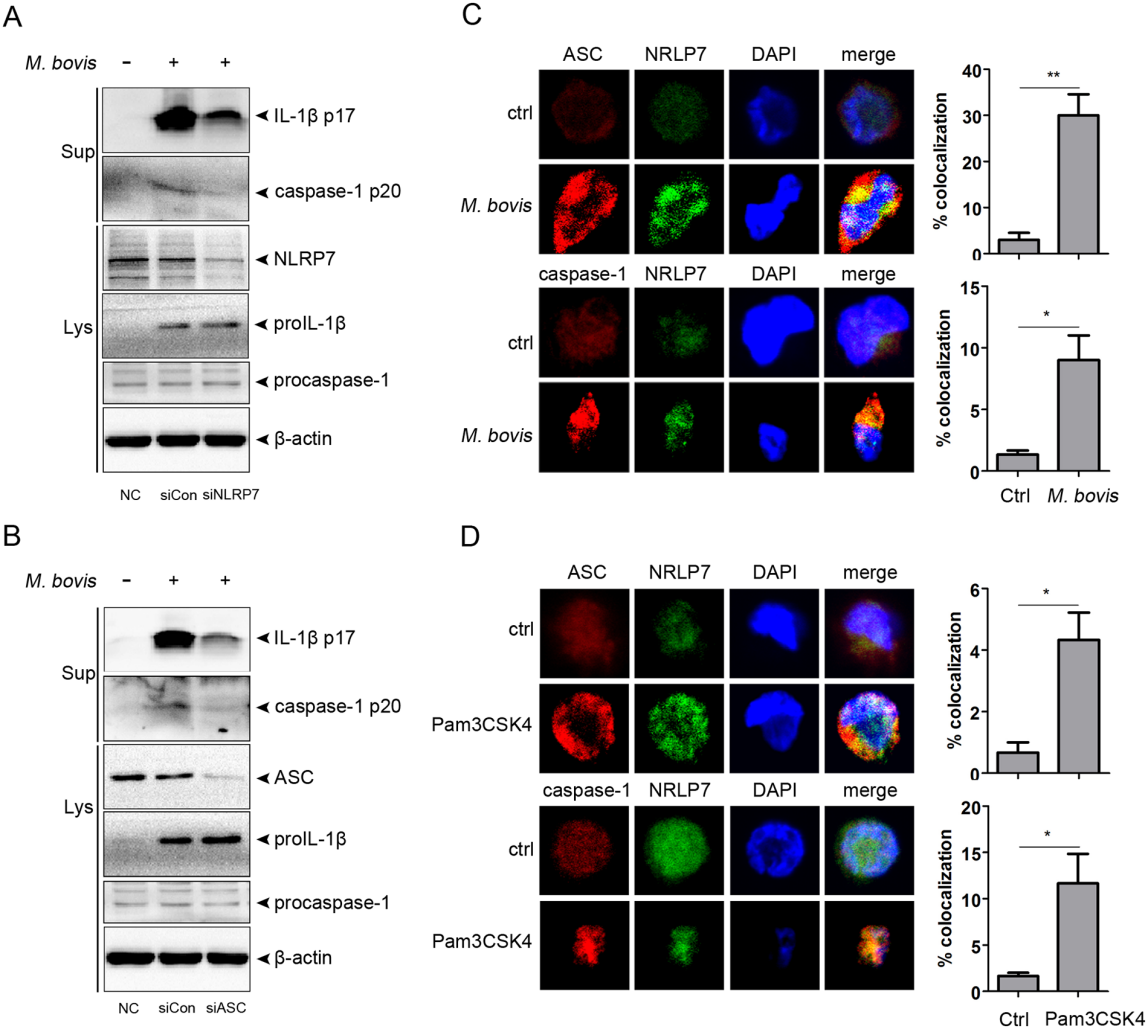
The NLRP7 inflammasome facilitates caspase-1 activation and IL-1β secretion upon *M*. *bovis* infection. A—B. THP-1 cells were transfected with siRNA that targets NLRP7 or ASC, and then infected with *M*. *bovis*. Supernatant and cell lysates were analyzed by immunoblotting. C—D. Cells were stimulated with *M*. *bovis* or Pam3CSK4 for 6 h. Colocalization of NLRP7 and ASC or caspase-1 was analyzed by confocal microscopy. Magnification, ×60.

To further investigate whether the NLRP7 inflammasome plays a role in *M*. *bovis*-induced IL-1β, we utilized a pool of siRNAs to knock down NLRP7 in THP-1 macrophages. Compared to non-targeting control, siRNA-mediated knockdown significantly reduced the protein levels of NLRP7, and also attenuated caspase-1 activation and IL-1β secretion following stimulation with *M*. *bovis* (Fig 3A). NLRP7 promotes IL-1β secretion via activation of the inflammasome, which is a multiprotein complex that contains ASC and caspase-1. siRNA knockdown experiments also showed that loss of ASC significantly reduced the induction of caspase-1 activity and IL-1β secretion following infection with *M*. *bovis* (Fig 3B).

Upon specific stimulation, NLRP7 colocalizes and interacts with ASC and caspase-1 to form the NLRP7 inflammasome. To confirm *M*. *bovis*-induced NLRP7 inflammasome activation, we carried out an immunofluorescence assay. In *M*. *bovis*-infected THP-1 macrophages, we observed that NLRP7 colocalized with ASC and caspase-1 in the perinuclear area, with some colocalization in the nucleus (Fig 3C). These effects were also observed after stimulation with Pam3CSK4, an NLRP7 inflammasome inducer (Fig 3D). Taken together, these data suggest that *M*. *bovis* infection induces NLRP7 inflammasome activation, which in turn promotes caspase-1 activation and IL-1β secretion.

### NLRP7 inflammasome activation induces *M*. *bovis*-mediated pyroptosis

**Fig 4:**
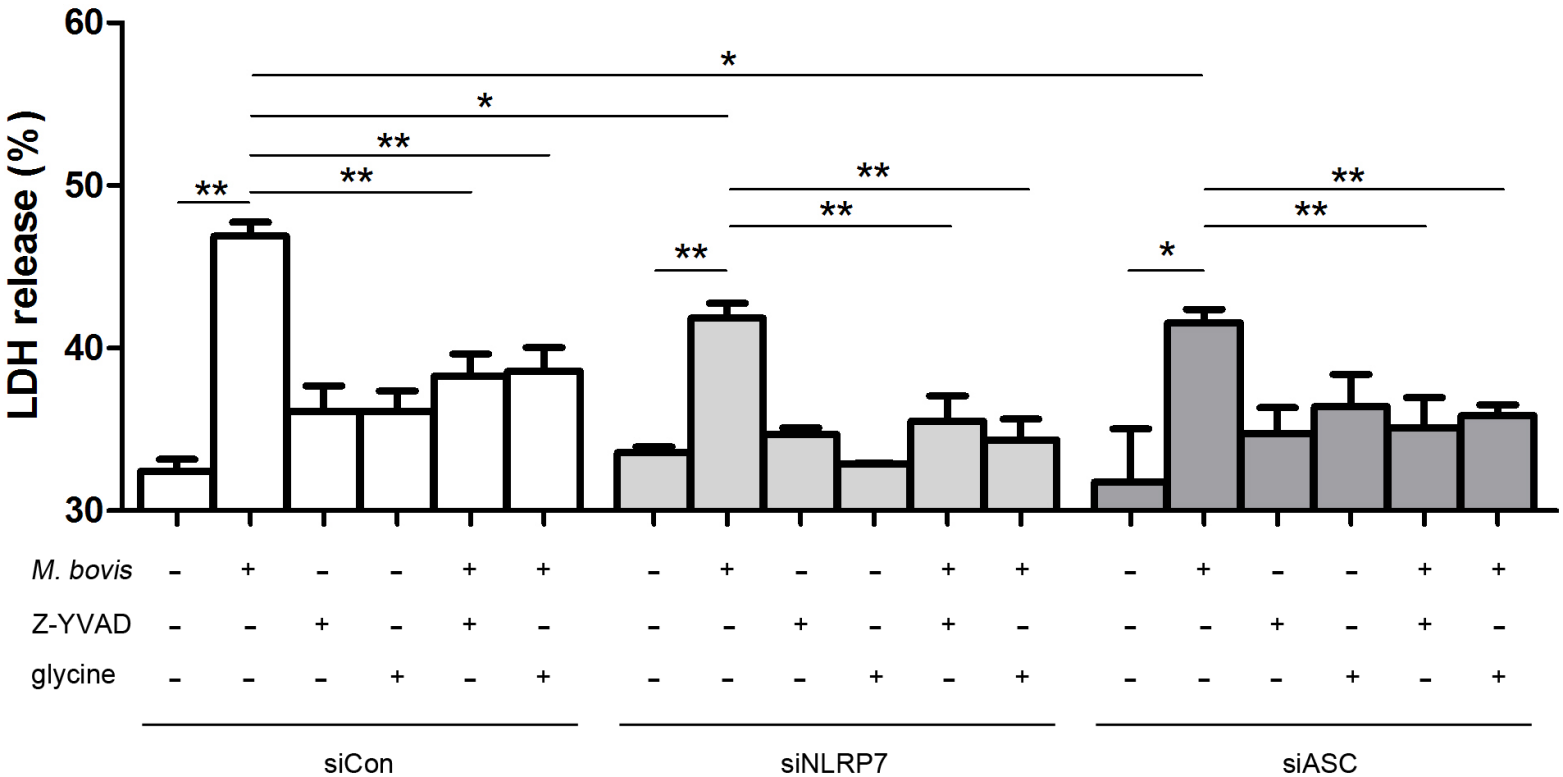
NLRP7 inflammasome activation promotes induction of pyroptosis in *M*. *bovis*-infected THP-1 cells. Cell death was evaluated by LDH release in NLRP7- or ASC-silenced cells stimulated with *M*. *bovis* in the presence or absence of Z-YVAD-FMK or glycine. Abbreviations: siCon, control non-targeting siRNA; siNLRP7, NLRP7-targeting siRNA; siASC, ASC-targeting siRNA.

Inflammasome activation is linked to caspase-1 dependent cell death called pyroptosis [23]. We evaluated cell death by LDH release, and observed that *M*. *bovis* infection led to significant increase of LDH release, and inhibition of caspase-1 bioactivity markedly decreased this effect (Fig 4). To evaluate whether NLRP7 inflammasome activation relates to pyroptosis, NLRP7- and ASC-silenced cells were stimulated with *M*. *bovis* in the presence or absence of the caspase-1 inhibitor, Z-YVAD-FMK. The results indicated that NLRP7 or ASC silencing attenuated LDH release, but made no difference in the presence of caspase-1 inhibitor compared to non-targeting control following infection, suggesting that NLRP7 inflammasome activation is involved in the cell death induced by *M*. *bovis* infection which is dependent on caspase-1 (Fig 4). Cell lysis during pyroptosis results from caspase-1-mediated pore formation in the cell membrane and subsequent influx of extracellular fluid [23]. The cytoprotective agent glycine inhibits pyroptosis because it nonspecifically prevents ion fluxes and suppresses swelling and lysis [24]. Addition of glycine to the culture medium substantially decreased LDH release in *M*. *bovis*-infected cells; this was not further enhanced by silencing of NLRP7 or ASC (Fig 4). Taken together, these data show that NLRP7 inflammasome activation contributes to pyroptosis induced by *M*. *bovis* infection.

### NLRP7 inflammasome activated by *M*. *bovis* promotes mRNA expression of IL-1β, TNF-α and CCL3, but inhibits IL-18 expression

**Fig 5:**
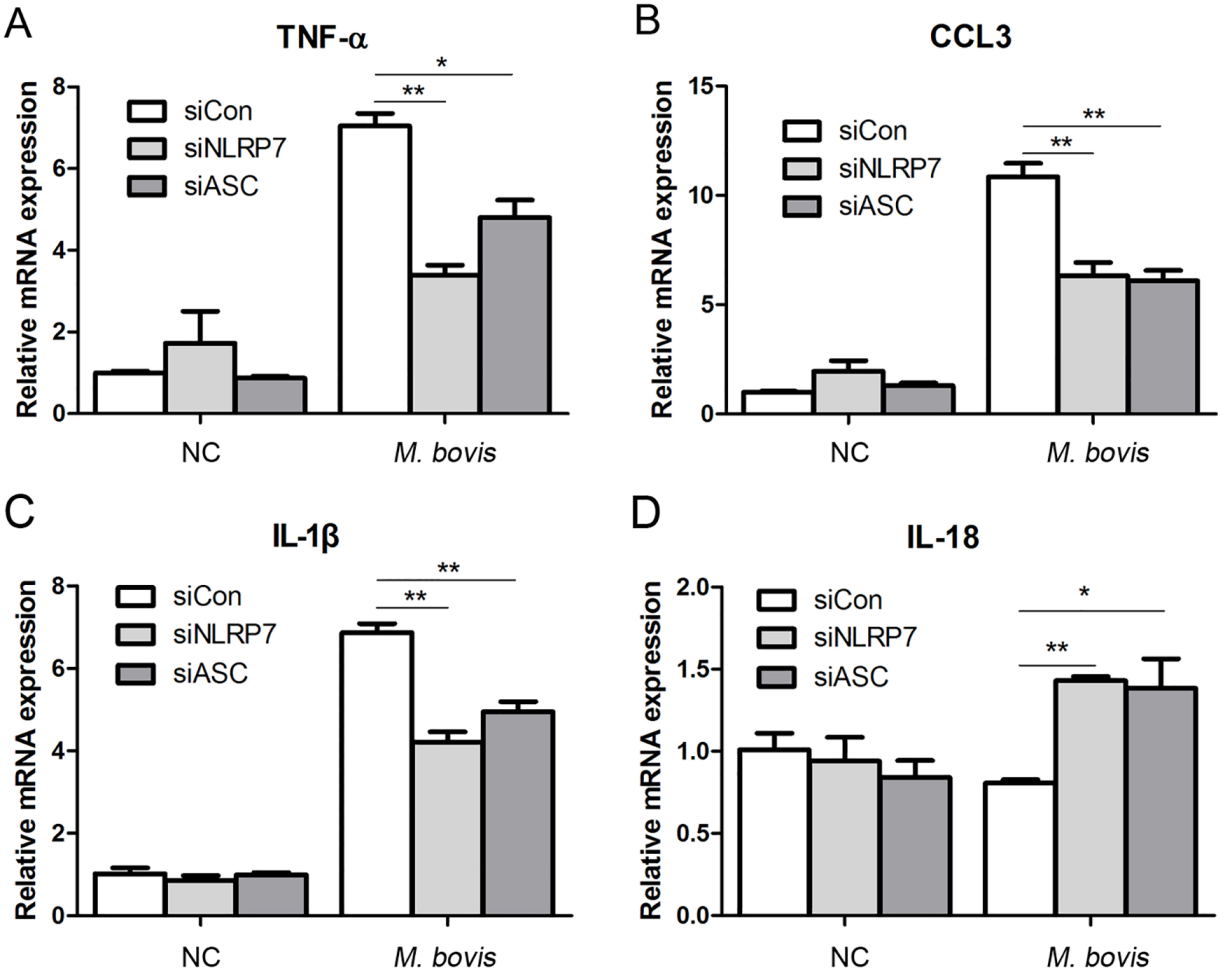
NLRP7 inflammasome activation influences the expression of TNF-α, CCL3, IL-1β and IL-18 mRNA. A—B. THP-1 macrophages were transfected with non-targeting siRNA, or NLRP7-targeting siRNA, or ASC-targeting siRNA, and then infected with *M*. *bovis*. Lysates were subjected to quantitative real-time PCR analysis.

Virulent mycobacteria causes a potent inflammatory response characterized by macrophage generation of cytokines, including TNF-α and CCL3, which contributes to granuloma formation through the recruitment of more macrophages and lymphocytes [25]. To investigate whether the NLRP7 inflammasome plays a role in the production of TNF-α and CCL3 besides IL-1β and IL-18, we silenced NLRP7 or ASC in THP-1 macrophages prior to infection, which significantly attenuated *M*. *bovis*-induced upregulation of TNF-α, CCL3 and IL-1β at the mRNA level (Fig 5A–5C). Additionally, *M*. *bovis* infection still led to obvious increases in IL-1β, TNF-α and CCL3 *mRNAs* in NLRP7- and ASC-silenced cells. However, *M*. *bovis* infection failed to change IL-18 mRNA expression, while NLRP7- or ASC-silencing led to its upregulation (Fig 5D), which may be due to various sample-collection times. These data suggest that the NLRP7 inflammasome is involved in *M*. *bovis*-induced upregulation of the mRNA expressions of TNF-α, CCL3 and IL-1β as well as downregulation of the mRNA expression of IL-18.

## Discussion

Previous studies described the interaction between virulent mycobacteria and their hosts. Virulent mycobacteria lead to the activation of NLRP3 inflammasome through recognition of the ESAT-6 protein [21], However, a later study suggested that NLRP3 inflammasome activation was dispensable for the control of pulmonary tuberculosis [26]. The AIM2 inflammasome is activated through the recognition of *M*. *tuberculosis* DNA in infected peritoneal macrophages [20], but Shah *et al*. demonstrated that *M*. *tuberculosis* inhibits AIM2 inflammasome activation in BMDCs [27]. Here we found that virulent *M*. *bovis* also activates the NLRP7 inflammasome in THP-1 macrophages, and contributes to induction of pyroptosis and expression of TNF-α and CCL3 due to NLRP7 inflammasome activation, although the precise mechanisms responsible for NLRP7 inflammasome activation in *M*. *bovis*-infected macrophages are still unclear.

The effect of NLRP7 on IL-1β secretion is debatable; the initial *in vitro* investigation by Kinoshita *et al*. linking NLRP7 to IL-1β secretion was based on inflammasome reconstitution assays in HEK293 cells, which displayed a negative regulation. NLRP7 inhibits processing of procaspase-1 and proIL-1β and LPS-induced IL-1β secretion through interaction with both of these proteins [28]. Another study by Khare *et al*. using stably silenced targeted gene in THP-1 cells proved that NLRP7 assembles with procaspase-1 and ASC to form the inflammasome, and contribute to increased IL-1β secretion in response to some heat-killed bacteria. Co-expression of NLRP7 with ASC, procaspase-1 and proIL-1β also led to increased level of active IL-1β release compared to the level of active IL-1β in the absence of NLRP7 [12], which is opposite to the results presented by Kinoshita *et al* [28]. In our study, we found that knockdown of endogenous NLRP7 in THP-1 macrophages led to reduced IL-1β secretion in *M*. *bovis*-infected cells. This is consistent with the data of Khare *et al*. [12], showing that NLRP7 positively regulates IL-1β release through inflammasome activation in response to *M*. *bovis* infection.

The mechanisms involved in inflammasome activation are complicated, with some bacteria and viruses activating more than one type of inflammasome due to their possession of different PAMPs. For example, *Listeria monocytogenes* induces activation of the NLRP3, AIM2 [29], NLRC4 [30], and NLRP7 inflammasomes within 16 hpi [12]. *M*. *bovis* infection caused upregulation of NLRP3, NLRP7 and AIM2 mRNAs at different time points, which may reflect the temporal production of specific bacterial inflammasome stimuli. Thus, the NLRP3 inflammasome is activated by *M*. *tuberculosis* as early as 4 hpi [26], and the role of AIM2 inflammasome in *M*. *tuberculosis*- and *M*. *bovis*-infected macrophages were detected at 24 hpi [19, 20].

NLRP7 was upregulated at the mRNA level, but not at the protein level in THP-1 macrophages. This may be similar to NLRP3, which is increased at the protein level in activated RAW 264.7 cells, primary cultured astrocytes of mice, and LPS-primed bone marrow macrophages [31–33], but not in LPS-treated THP-1 cells [34]. The reason for these differences may be due to cell type or to the effect of PMA on protein expression [35].

Pyroptosis, a type of programmed cell death which is dependent on caspase-1, is an efficient mechanism of intracellular bacterial clearance [36]. Here, we found that the NLRP7 inflammasome plays a role in the induction of pyroptosis in *M*. *bovis*-infected cells. Although proinflammatory cytokine release and pyroptosis are both induced by *M*. *bovis* infection, and require caspase-1 activation, these two processes may occur independently, and previous studies demonstrated that *Legionella pneumophila*, *Burkholderia thailandensis* and *Salmonella typhimurium* that persistently expresses the flagellin protein were cleared through the pyroptosis pathway and independently of cytokine release [36].

Granulomas are typical histopathological changes in tuberculosis, which represent a ‘stalemate’ between the host and the bacteria, benefitting both parties [37]. TNF-α and chemokines play a vital role in granuloma formation and facilitate restriction of *M*. *tuberculosis* infection [38, 39]. In this study, *M*. *bovis*-induced activation of the NLRP7 inflammasome promoted the expression of the TNF-α and CCL3. Silencing of NLRP7 or ASC did not block the induction of TNF-α in *M*. *bovis*-infected THP-1 cells, which may be due to incomplete silencing, and/or the existence of alternate pathways such as NF-κB signaling [40]. For example, avirulent *M*. *bovis* BCG is unable to activate the inflammasome in the infected macrophages [26], but it still increases the transcription of TNF-α [41].

In conclusion, we demonstrate that *M*. *bovis* infection activates the NLRP7 inflammasome, which in turn facilitates IL-1β secretion, induction of pyroptosis, and upregulation of TNF-α, CCL3 and IL-1β at the mRNA level. Our study contributes to a better understanding of innate immune response to mycobacterial infection.
